# The Pattern of Fruit and Vegetable Consumption among Saudi University Students

**DOI:** 10.5539/gjhs.v6n2p155

**Published:** 2013-12-24

**Authors:** Hala Hazam AL-Otaibi

**Affiliations:** 1 Department of Food Sciences and Nutrition, Community Nutrition, College of Agriculture and Food Science, King Faisal University, Al-Ahsa, Saudi Arabia

**Keywords:** fruit, vegetable, university students, self efficacy

## Abstract

The positive benefits of consumption fruit and vegetable are well documented in studies of health and body weight maintain. A cross sectional study was conducted utilized street based survey among 960 female students at King Faisal University in AL-Hasa, Saudi Arabia, to investigate the daily consumption of fruits and vegetables and the psychosocial factors related to the consumption. Seventy-eight percent of students consuming <5 servings/day of fruit and vegetable with only 22% of them consuming ≥5 servings/day, majority of them are in the normal BMI category. For psychosocial factors the higher consumption group more knowledgeable about the daily consumption of fruit and vegetable, and had more confidence in eating fruit and vegetables under difficult circumstances with significant differences between the groups (P=0.000, P=0.045; respectively). Self efficacy was significantly predictor for recommended daily fruit and vegetable consumption (β=0.303, SE=0.023, P=0.000), but perceived barriers and knowledge not predictor for recommended daily consumption. The results of this study can be useful to design an intervention to promote fruit and vegetable intake among this target group.

## 1. Introduction

A diet high in fruit and vegetables is recommended for men and women to reduce the risk of many chronic diseases (Ness & Powles, 1997; Gillman et al., 1995; Steinmetz & Potter, 1996) and maintain a healthy body weight (WHO, 2005). The WHO recommendations encouraging adults to consume at least five servings or 4000 g of fruit and vegetable daily (WHO, 2005), also in Saudi Arabia, a number of dietary recommendations and guidelines have been developed by the Ministry of Health such as the Healthy Palm Saudi dietary guidelines (Alnakhala Elgethaiya) with the aim to encouraging the individuals to eat healthier and to consume three to five servings of vegetables and two to four servings of fruits per day (MOH, 2013).

Several studies indicate that consumers in many age groups in Saudi Arabia ingest an adequate amount of fruit and vegetable daily (AL-Qauhiz, 2010; Abdel-Megeid, Abdalharem, & EL- Fetouh, 2011), especially in university students. Usually the transition from adolescence to young adulthood is a period often characterized by an unhealthy lifestyle in which, younglings become independent and adopt lasting health behavior patterns (Huang et al., 2003; AL-Qauhiz, 2010; Abdle-Megeid et al., 2011).

AL-Qauhiz (2010) reported only 17.2% from 799 female university students incross sectional study at Saudi consumed fruit and vegetable daily, also Aboul Azm and Elebiary (2010) found that 30% of the female nursing student in Saudi Arabia ate fruit and vegetable daily. Some of the individual factor include psychosocial such as knowledge (Watters et al., 2007), barriers (Steptoe et al., 2003) and self efficacy (Chunga & Hoerr, 2005), found to be a factor affecting fruit and vegetable intake and strong predictors for the consumption (Watters et al., 2007; Steptoe et al., 2003; Chunga & Hoerr, 2005; Van Duyn et al., 2001). Due to our knowledge no previous study conducted among female university students in Saudi Arabia examined the relationship between the psychosocial factors and daily consumption of fruit and vegetable. The present study examined the daily consumption of fruits and vegetables and the psychosocial factors related to the consumption among female university students in AL-Hasa, Saudi Arabia.

## 2. Methods

### 2.1 Study Design and Subjects

Present study is a cross sectional study was conduct utilizing a street based survey among convenience 960 female student’s non- food/nutrition major at King Faisal University in AL-Hasa, Saudi Arabia during the second semester of 2013. The study conducted among female only because it is not allowed for female researchers to access to male campus according to Saudi community traditions, were male and female campuses separated. Sample size based on Daniel et al. (1999) sample size formula (n=z^2^pq/d^2^), the required sample for this study was 324 students, where n is the minimum sample size, z= standard deviation score =1.96, p= prevalence of daily consumption of fruit and vegetables 30% among university students (Aboul Azm & Elebiary 2010), q=1-p, d= 0.05 at the 95% confidence interval. n=z^2^pq/d^2^, n= (1.96)^2^x (0.3) x (1-0.3)/(0.05)^2^ =324students.

Ten undergraduate female students in the food and nutrition major volunteered to collect the data. The times and locations were chosen based on the times of the day that classes were offered and the locations as cafeterias and libraries in the colleges, main library and bus stops (female campus), where the students are usual gather to increase the probability of obtaining a representative sample of the undergraduate students. After taking the necessary approvals from college of Agriculture and Food Science, our questioners distributed among students after briefing them about the purpose of the study. All the students were Saudi, disease free and not pregnant.

### 2.2 Instrument

Face and content validity of the survey were determined through a pilot study on 30 students that were not included in the study. Because of limited contact time with students, the survey was limited to a two-page instrument. The volunteers were equipped with a set of questionnaire tool (cups, bowls, images of serving dishes, spoons and photos of vegetable and fruit) to help the students to estimate fruit and vegetable serving. Demographic information and anthropometric measurements as height and weight were self report by the students, and then we calculated the Body Mass Index (BMI) and categorized according to the World Health Organization guidelines (WHO, 1998).

Students were asked in two separate questions: How many serving of fruits consumed each day?; How many serving of vegetable consumed each day?. The five response categories were: none, one serving, two serving, three to four serving and five serving or more. The two questions have been previously shown to be an indicator of fruit and vegetable consumption and theses questions have been validated in may studies (Staser et al., 2011; Welch et al., 2008; Ball et al., 2006). The Cronbach’s alpha co-efficient for the two items was 0.871. Alpha equal to or greater than 0.7 was considered satisfactory. Six items of knowledge in fruit and vegetable knowledge scale was modified from an existing scale (Holdsworth et al., 2006; Turrell, 1997). The Cronbach’s alpha co-efficient for the six items was 0.895. Respondents answered ’’true, false, and I don’t know’’, the maximum scores six and the minimum zero. Perceived barriers of consumption fruit and vegetable scale consisted of seven items modified from previous study (Townsend & Kaiser, 2005). The format of the scale included the three Likert scale, maximum scores 21 and the minimum seven The Cronbach’s alpha co-efficient for the seven items was 0.863. Self-efficacy for eating fruit and vegetables was assessed by five items on a 3-point Likert scale the total possible score were 15 and the minimum score five. Self-efficacy items modified from previous studies (Henry et al., 2006; McAThur & Pawlak, 2011). The Cronbach’s alpha co-efficient for the five items was 0.763.

### 2.3 Data Analyses

The analysis was conducted using SPSS 19 with statistical significance level set at p <0.05. First, the socio-demographic characteristics, anthropometric measurements, knowledge, barriers and self-efficacy of the sample were presented by fruit and vegetables consumption ≥5 servings and <5 servings for differences using independent T-test and chi-square test. Second, the linear regression analysis was conducted to examine predictors of the recommended daily intake of fruit and vegetable.

## 3. Results

Demographic data were shown in Table 1. Only 210 (22%) of the students consuming five serving of fruit and vegetable daily, the mean age forthem is 21 years, and most students in ≥5 servings/day group (62.5%) had family income above 30000 Riyal, with a significant difference between the groups (*P*=0.004). Majority of the students were single in both groups. Students in the <5 serving /day group had overweight mean BMI (25.7±4.3) and ≥5 servings/day group had normal mean BMI (kg/m^2^) (23.1±5.3) with significantly different between the groups. According to BMI (kg/m^2^) categories almost similar percentage in ≥5 servings/day group and <5 servings /day group (20, 18.7%, respectively) were overweight, while 80% were within the normal BMI range in the ≥5 servings/day group. Low proportion (2.2%) of the <5 servings /day group were obese and underweight (8.8%) (Table 1). Twenty two percent of them ate six servings of fruits and vegetables daily (Table 1), and the rest (<5 servings/day group) consumed almost three servings/day of fruit and vegetable which are fewer than the minimum recommendation with a significant difference between groups.

**Table 1:** Demographic characteristics, anthropometric measurement and fruit and vegetables intake of students

	<5 servings/day n=750(78%)	≥5 servings/day n=210(22%)	*P*
*Demographic characteristics*			
Age (years)	21.81±1.78	21.67±1.32	^a^0.069
Marital status			
Single	395 (52.7%)	126 (60%)	^b^0.078
Married	355 (47.3%)	48 (40%)	
Family income (SR^c^)				
<3000 SR	190 (25.3%)	-	^b^0.004*
3001-5000 SR	222 (29.7%)	131 (62.5%)	
5001-10,000 SR	223 (29.7%)	37 (17.5%)	
>10,000 SR	115(15.4%)	42 (20%)
*Anthropometric measurement and indicators*			
Weight (kg)	63.84±12.76	56.42±15.24	^a^0.001**
Body mass index (BMI) (kg/m^2^)	25.75±4.32	23.19±5.39	^a^0.002*
Underweight (<18.5)	66 (8.8%)	-	^b^0.000**
Normal weight (18 -24.9)	527 (70.3%)	168 (80%)	
Overweight (25-29.9)	140 (18.7%)	42 (20%)
Obese (<30)	17 (2.2)	-
*Fruit & vegetable intake* (servings)			
Fruit	1.71±0.84	3.1±1.43	0.000**
Vegetables	1. 12±1.12	3.21±1.32	0.000**
Fruit & vegetables	2.83±1.44	6.31±2.75	0.000**

* *P*<0.05,

** *P*<0.001,

a T-test,

b χ^2^,

c Saudi Real, 1 USD = 3.75 SR.

Higher (4.34±1.37) and significant (*P*=0.045) mean Knowledge of fruit and vegetable intake was reported to students in ≥5 servings/day group comparable to <5 servings/day group (3.4±1.79), furthermore they are knowledgeable about the recommended daily intake of fruit and vegetable, obesity and of fruit and vegetable intake, and the damage happens for nutrients results of overcook with a significant difference between groups. The higher consumption group had higher mean knowledge in the rest of statements but without significant difference between groups (Table 2). However, for barriers the <5 servings /day group have more mean barriers to know enough recipes for fruit and vegetable, and they believe that fruit and vegetable are expensive and not delicious; also it is difficult to them eat five servings daily, they don’t have time to prepare vegetables as part of their diet, and they cannot purchase fruit and vegetables in restaurant or university comparable to the ≥5 servings/day group, but without significant difference in the total barriers despite the ≥5 servings/day group had low mean score in three items and total barriers (Table 3). Table 4 shown that the ≥5 servings/day group had higher total mean of self efficacy score and more confidence to four items with significantly different between the groups (*P*=0.000). Self efficacy (β=0.303, SE=0.023, *P*=0.000) was significantly a predictor for recommended daily fruit and vegetables consumption, but greater level of knowledge (β=0.028, SE=0.028, *P*=0.378) and lower perceived barriers (β=0.055, SE=0.019, *P*=0.101) were not predictor to the recommended daily consumption of fruit and vegetable (Table 5).

**Table 2:** Knowledge of fruit and vegetable intake (Mean ± SD)

Knowledge	<5 servings/day n=750	≥5 servings/day n=210	*P*
5 servings of fruit & vegetables should be eaten every day for preventing major diseases.	0.37±0.12	1±0.41	0.009*
Low intake of fruit & vegetables can contribute to heart problems.	0.58±0.23	0.6±0.41	0.86
Low intake of fruit & vegetables can contribute to obesity.	0.71±0.42	1±0.1	0.000**
Low intake of fruit & vegetables can contribute to certain cancers.	0.53±0.43	0.6±0.32	0.39
It will help me to get enough vitamins and minerals.	0.54±0.32	0.6±0.41	0.203
Overcook influences the availability of nutrients in fruit & vegetables.	0.21±0.1	0.54±12	0.006*
Total knowledge	3.4±1.79	4.34±1.37	0.045*

* *P*<0.05,

** *P*<0.001

**Table 3:** Barriers of fruit and vegetable intake (Mean ± SD)

	Barriers	<5 servings/day n=750	≥5 servings/day n=210	*P*
1-	It is difficult to eat 5 servings of fruit & vegetables every day.	2±0.9	1.72±0.61	0.007*
2-	Preparing and cooking vegetables would be time consuming.	2.4±0.49	2.05±0.66	0.000**
3-	I don’t know enough recipes for fruit & vegetables.	1.8±0.44	1.64±0.73	0.147
4-	Fruit & vegetables are expensive.	2.6±0.49	2.4±0.63	0.078
5-	Fruit & vegetables are not delicious.	1.55±0.71	1.45±0.49	0.305
6-	Members of my household won’t eat fruit & vegetables.	2.34±0.72	2.21±0.75	0.186
7-	I can’t get fruit & vegetables in restaurant and university.	1.56±0.71	1.2±0.4	0.000**
	Total barriers	13.6±0.8	13.27±2.7	0.406

* *P*<0.05,

** *P*<0.001

**Table 4:** Self-efficacy of fruit and vegetable intake (Mean ± SD)

Self-efficacy	<5 servings/day n=750	≥5 servings/day n=210	*P*
Eat fruit & vegetables as part of lunch most days.	1.81±0.8	2.82±0.41	0.000**
Eat fruit & vegetables for a snack instead of chips or candy.	2.12±0.75	2.6±0.49	0.001**
Eat fruit & vegetables when eating out at restaurant and university.	2.25±0.73	3.0±0.12	0.000**
Eat 5 servings of fruit & vegetables every day.	1.28±0.54	2.5±0.81	0.000**
Drink 100% fruit juice instead of soda or fruit punch.	1.97±0.83	2.2±0.75	0.067
Total self-efficacy	9.54±2.11	13.12±2.1	0.000**

*P<0.05,

** *P*<0.001

**Table 5:** Linear regression analysis predicting of fruit and vegetables intake

Model	B	S.E	β	t	P
Constant	0.447	0.272	-	1.641	0.101
knowledge	0.025	0.028	0.028	0.883	0.378
Barriers	0.032	0.019	0.055	1.643	0.101
Self-efficacy	0.211	0.023	0.303	9.074	0.000**
**R^2^=0.113; adjusted R^2^=0.110**					

*P<0.05,

** *P*<0.001

## 4. Discussion

Majority of the students (78%) of had low consumption from fruit and vegetable and only 22% consumed the recommended daily intake, which is similar to research conducted among female university students in Saudi Arabia. Abdle-Megeid et al. (2011) reported a higher percentage (40%) among female student’s food/nutrition major were students concern about their health. Research in other countries as Musaiger et al. (2011) found that 26.3% of Bahrain students consumed five servings of fruit and vegetable daily, while 20% of Pakistani female university students consumed only two serving daily of fruit and vegetable (Khalid et al, 2011). Western countries such as German (Keller et al., 2008)), Britain (Dodd et al., 2010) and US (King et al., 2007) reported between 66% and 95% of university students, not meeting fruit and vegetable consumption recommendations which similar to present study percentage (78%).

In contrast, many studies conducted among female university students in South East Asia and Mediterranean countries proved higher percentage of fruit and vegetable consumption as 80% of Chinese students (Sakamki et al., 2005) 100% in Sir Lanka (Perera & Madhujith, 2012), 51% in Lebanon (Yahia et al., 2008), 40% in Turkey (Unusan, 2006), consumed five servings of fruit and vegetables daily. In general all, the research conducted in South East Asia and Mediterranean region reflect the feeding habit in these regions South East where the traditional dishes are rich in fruit and vegetable ingredients. Research conducted in Saudi Arabia found university less than half of students are eating at least five servings daily, while these results may represent the feeding habit in Saudi culture while eating raw fruit and vegetables in the course of a meal is uncommon among Saudi people; also fruits are usually taken as desserts after meals and that can explain why student in <5 servings /day group consumed almost two servings of fruit. However, the traditional meal (Kabsa) of Saudi contain small amount of vegetable (rice, lamb meats, onion and tomato).

Previous research has been well documented the impact of low fruit and vegetable consumption on BMI and show that the less fruits and vegetables in adults diet cause to be overweight or obese (Bates et al., 2008; Davis et al., 2006), our results found that <5 servings /day group had overweight mean of BMI and almost one fifth of students were in overweight category comparable to ≥5 servings/day group were 80% of them in the normal BMI category. Overall young female students usually worried about their body shape (AL-Otaibi et al., 2013), and that explain why obesity and overweight did not prevalent among the groups.

Nutrition knowledge is one of the key factors to improving eating behavior for health and overall wellbeing in adults (McCullough et al., 2002). The present study found the ≥5 servings/day group scored significantly higher mean of total knowledge scale than the <5 servings /day group, but without significant different between groups which are similar to Kolodinsky and Colleagues (2007) who conducted internet survey among university students in Virginia and reported students who consumed five serving daily or more had higher mean of total knowledge related to fruit and vegetable consumption than students who consumed less than five serving daily but without significant difference (*P* ≥0.05). Overall, the <5 servings /day group did not attain the recommended level of consumption which is similar to a cross sectional study among university nursing students in South Africa the researcher found only 44.7% of the students know the daily recommended number of servings of fruit and vegetable (Violet et al., 2012). Both groups in present study had moderate mean of knowledge about the relationship between cancers, heart problems and low intake of fruit and vegetable, but Shive et al, (2003) reported lower mean (mean=2.79±0.88 out of five points) about the relationship between diet and cancers and diseases among female university students comparable to the present study.

As the present study some research conducted among young female found time pressure barrier for fruit and vegetables consumption, while King et al. (2007) found more than half (57.8%) of female university students reporting time as barrier to eat fruit and vegetables, also Welch et al. (2008) reported that 54% of female under 30 years old time was barrier to them to eat fruit and vegetables. This highlights the importance of developing intervention that focus on improving time management skills among students. However, both groups reported higher mean for statement members of their household won’t eat fruit and vegetable, while majority of them unmarried and living in the family house eat what their mothers prepared for the family food, in Saudi Arabia majority of the families still gathered to eat the three major meals (AL-Oboudi & AL-Amer, 2006). While, we thought both groups had limited cooking skills, because they do not know enough recipes to prepare a basic, healthful meal. Finally taste was not barriers for both groups, but for price they believe fruit and vegetables are expensive. Other studies reported both taste and price were barriers for female university students (King et al., 2008; Shive et al., 2003).

The results of the current study indicate that self-efficacy has a significant positive effect on the number of fruits and vegetables eaten by the students, while they are more confidence in eating fruit and vegetables under difficult circumstances, which are agreement with similar studies conducted among female (Chung & Hoerr, 2005; Steptoe et al., 2009; Henry et al., 2006). However, self-efficacy was confirmed as a positive predictor for fruit and vegetable intakes in the present study as other studies have shown. Henry and Colleagues (2006) reported self-efficacy was predictor for higher consumption of fruit and vegetables in low income African American mothers, also Chung and Hoer (2005) found that self efficacy predicted to eat fruit and vegetables among 236 female university students in US (*P*<0.001). Knowledge and barriers of fruit and vegetables consumption was not predictor for intakes in the present study, Sharma et al. (2008) conducted cross sectional study to examine the association between nutrition knowledge and eating behavior in Mexican American and they found nutrition knowledge was not significant predictor for fruit and vegetable intakes. Furthermore, a literature review by Worsely (2002) reported that nutrition knowledge had very small impact on fruit and vegetable intake.

There are some limitations in the present study. First, this study design (cross sectional) does not allow the establishment of causal relationship. Second, the students might not be representative for a larger population of young adults in Saudi Arabia. Third, the anthropometric measurements were self-reported, it is entirely possible that some students did not know how much they currently weigh or did not want to share weight. Fourth, the street based survey nonrandom sample and that there was no opportunity for any follow up questions. Finally, fruit and vegetable intake were assessed only in two short separate questions may cause the under or overestimation of intake. However, this tool was validated in a sample group which had the same characteristics as the students. The two questions has been previously validate in many studies (Staser et al., 2011; Welch et al., 2008; Ball et al., 2006). These limitations notwithstanding the present study also have strengths. It is a street based survey is short data collection time, the low cost and involvement of undergraduate students in research project. In addition, there is an assurance that the student who received the questionnaire actually completed the questionnaire, and there are no opportunities to provide assistance with answering the questions.

Overall, this study highlights the role of self efficacy in fruit and vegetable consumption for female students. The results of this study can be useful to design intervention to promote fruit and vegetable consumption among this target group.
